# Screening and systematic follow-up for cardiopulmonary comorbidity in elective surgery for colorectal cancer: a randomised feasibility study

**DOI:** 10.1186/s12957-019-1668-7

**Published:** 2019-07-22

**Authors:** Hans B. Rahr, Susanna Streym, Charlotte G. Kryh-Jensen, Helene T. Hougaard, Anne S. Knudsen, Steffen H. Kristensen, Ejler Ejlersen

**Affiliations:** 1 Department of Surgery, Vejle Hospital, University Hospital of Southern Denmark, DK-7100 Vejle, Denmark; 2 Colorectal Cancer Center South, Vejle Hospital, University Hospital of Southern Denmark, DK-7100 Vejle, Denmark; 3 OPEN—Open Patient Data Exploratory Network, Odense University Hospital, DK-5000 Odense, Denmark; 4 Institute of Regional Health Research, Vejle Hospital, University of Southern Denmark, DK-7100 Vejle, Denmark; 5 Department of Cardiology, Vejle Hospital, University Hospital of Southern Denmark, DK-7100 Vejle, Denmark; 6 Department of Medicine, Vejle Hospital, University Hospital of Southern Denmark, DK-7100 Vejle, Denmark

**Keywords:** Colorectal cancer, Comorbidity, Risk assessment, Postoperative complications

## Abstract

**Background:**

One third of patients with colorectal cancer (CRC) have comorbidity, which impairs their postoperative outcomes. Scoring systems may predict mortality, but there is limited evidence of effective interventions in high-risk patients. Our aim was to test a trial setup to assess the effect of extra postoperative medical visits and follow-up on 1-year mortality and other outcomes in patients with cardiopulmonary risk factors undergoing elective surgery for colorectal tumours.

**Methods:**

Patients preoperatively screened positive for cardiopulmonary comorbidity were eligible. On postoperative day 4, they were randomised to either routine follow-up (RFU) or RFU with one extra medical visit and additional visits to the Cardiology and Respiratory Medicine Clinics 1 and 3 months postoperatively. The primary outcome measure was 1-year mortality; secondary outcome measures were length of stay (LOS), complications, and readmissions.

**Results:**

Of 673 screened patients 326 (48%) were found eligible, 108 declined participation, and 198 were randomised. Postoperative medical problems and/or need for intervention were found in 15–23% of the patients at the extra medical visits. The 90-day mortality was 0 and the 1-year mortality only 2.6% with no differences between the two groups. LOS and complication rates did not differ, but there were significantly fewer readmissions in the intervention group.

**Conclusions:**

The 1-year mortality after elective CRC surgery was low, even in the presence of cardiopulmonary risk factors. There was no evidence of reduced mortality with additional medical follow-up in these patients.

**Trial registration:**

ClinicalTrials.gov NCT02328365 registered 31 December 2014 (retrospectively registered)

## Background

Comorbidity has come into focus as a risk factor for adverse outcomes in cancer treatment. In colorectal cancer (CRC), approximately one third of the patients have significant comorbidity, and this impairs their survival [1–5]. While scoring and assessment systems may predict risk in the individual patient [6–13], there is limited data on effective interventions to improve the outcome in patients at risk, and most previous studies have focused on preoperative rather than postoperative interventions [14–21]. Colorectal surgery is a challenge to physiological homeostasis and entails substantial morbidity [22]. We hypothesised that adding extra medical visits to the postoperative follow-up of patients at risk would improve survival and other outcomes by ensuring that relevant medical problems are detected and managed adequately and that the patient’s overall condition is restored to an optimum. We decided to focus on cardiopulmonary comorbidity, which has been identified as an independent risk factor for postoperative death [2, 23].

The aim of this study was to test a trial setup with systematic preoperative screening for cardiopulmonary comorbidity and postoperative randomisation of eligible patients to either routine follow-up or routine follow-up with extra medical visits as a means to improve the outcome of surgical treatment for CRC. It was a separate goal of the study to obtain reliable estimates of the main outcome measures to form the basis of a future large-scale randomised trial.

## Methods

### Setting

The setting is a 34-bed surgical oncology unit receiving 250–300 new CRC cases yearly. Perioperative care follows the ERAS (Enhanced Recovery after Surgery) principles and national guidelines for treatment of colorectal cancer. Safe Surgery checklists and early warning scores are routinely applied as is a simple, systematic risk stratification system allocating patients to adequate levels of observation and care. The study was registered with ClinicalTrials.gov (NCT02328365).

### Design

We chose a randomised controlled design, but decided to begin with a feasibility study instead of a full-scale clinical trial, since it was unknown which rate of 1-year mortality to expect with the selection criteria and interventions used. Lacking a reliable mortality estimate on which to base sample size calculations for a group comparison, we decided to include enough patients to be able to estimate a mortality rate of 30% with a confidence interval of ± 10%, or at least 90 patients in each randomisation group.

### Patients

All patients scheduled for elective operation of verified or suspected CRC were screened by a study nurse for cardiopulmonary comorbidity at the preoperative visit. Inclusion criteria were based on national and local recommendations (Table 1). For a preoperative baseline evaluation, patients fulfilling the cardiologic criteria were referred to the Cardiology Clinic (CC) unless they had recently been there, and patients with pulmonary criteria were similarly seen in the Respiratory Medicine Clinic (RMC) for examination and spirometry. For ethical reasons, all patients screened positive were offered these visits regardless of participation in the study. Patients fulfilling at least one inclusion criterion could be included after written and orally informed consent provided they were 18 years or older, legally competent, able to comprehend the information, and did not have disseminated cancer with limited life expectancy. The study was approved by the Regional Committee on Health Research Ethics (S-20130132).

**Table 1:** Eligibility criteria used for screening 673 patients for cardiopulmonary comorbidity before planned colorectal cancer surgery

	Number of patients with positive criterion
Cardiovascular criteria^1^	
Ischaemic heart disease	66
Heart failure	19
Cerebrovascular disease	57
Insulin treatment	27
P-Creatinine level > 170 μmol/l	4
MET score 4 or less	36
Valve disorder	35
Hypertension > 180/100 mmHg	6
Significant arrhythmia	30
Number of cardiovascular criteria per patient (*n* = 179)	
1	108 (60%)
2	50 (28%)
3+	21 (12%)
Pulmonary criteria	
Regular medication for pulmonary disease	54
MRC^2^ dyspnoea score 3 or more	24
> 2 pulmonary infections treated with antibiotics within 12 months	10
20 or more pack-years of smoking	197
Number of pulmonary criteria per patient (*n* = 225)	
1	177 (79%)
2	38 (17%)
3+	10 (4%)

^1^For definitions, see [24]

^2^For definitions, see [25]

### Interventions

**Fig. 1:**
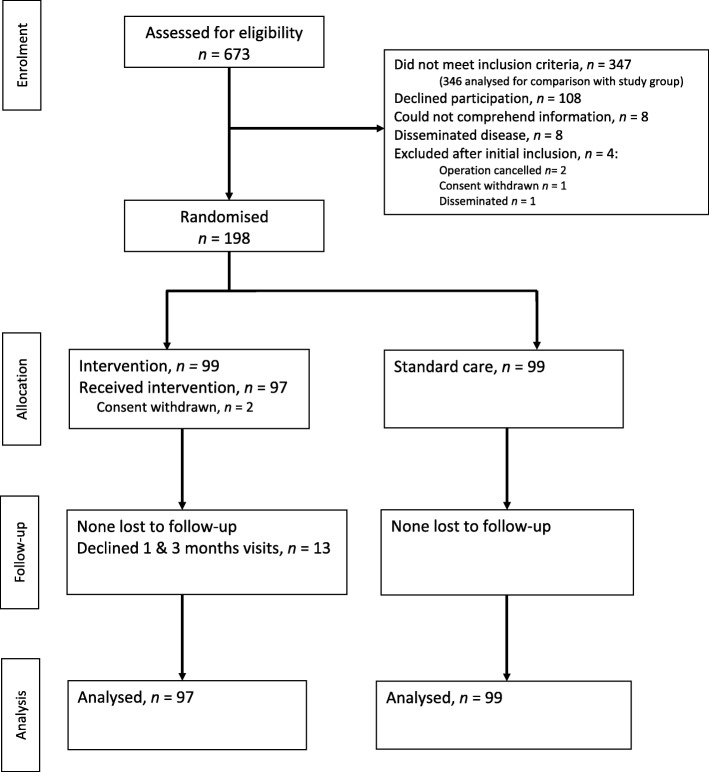
CONSORT flow diagram

Surgical treatment and perioperative care was delivered according to departmental guidelines. Included patients were randomised on postoperative day 4 (POD4) to either standard follow-up alone (“standard group”, SG) or standard follow-up plus extra medical visits and follow-up (“intervention group”, IG) (Fig. 1). IG patients were examined on POD4 or POD5 by an experienced physician from the Department of Medicine. Randomisation was performed in an online programme based on randomly permuted blocks of 2, 4, or 6 patients, and the result was kept concealed until the morning of the visit. The IG was also seen in the outpatient clinic 1 month (in CC and RMC) and 3 months (in RMC) after the operation.

### Data sources

All screened patients were entered into an inclusion log. Prospective clinical data were extracted from the electronic medical records (EMR) and entered into a custom-built database on the REDCap platform. Only observations and events documented in the EMR or at the screening interviews were recorded. Clinical events and outcomes were reviewed and validated by the senior authors. Complications were classified according to the Danish Colorectal Cancer Group (DCCG), i.e. as medical (stroke, acute coronary syndrome (ACS), aspiration, pneumonia, heart failure, arrhythmia, pulmonary embolism, lung failure, kidney failure, sepsis, deep vein thrombosis (DVT), arterial embolism, other), or surgical (bleeding, burst abdomen, obstruction/ileus, wound abscess, intraabdominal abscess, stoma complication, anastomotic leakage, other), and graded according to Clavien-Dindo [26, 27]. Since the Clavien-Dindo scale is designed specifically for surgical complications, we also graded the in-hospital medical complications by a Medical Event Severity Score (MESS) according to their consequences—grade 0, no intervention; 1, change of medication; 2, other specialist summoned; 3, transfer to other department; 4, transfer to ICU; and 5, death.

Additional clinical and pathology data on patients with malignant tumours were extracted from the national bowel cancer database (NBCD) containing prospective data on virtually all (98.6% in 2016) Danish patients with primary adenocarcinoma of the colon or rectum [28]. Danish surgical and pathology departments are required by law to enter the data in the NBCD, which is further enriched with data on comorbidity from the national patient registry (LPR), pathology data from the national pathology database (LRP), and mortality data from the Central Office of Civil Registration (CPR). NBCD is validated against LPR and LRP to ensure completeness. Data on length of stay (LOS), readmissions, and pre- and postoperative adjuvant treatment were obtained from the Region’s patient administrative database. The common key to all datasets was the individual civil registration number assigned to all Danish citizens.

### Data analysis

Results were reported according to CONSORT 2010 [29]. The primary outcome measure was the rate of 1-year mortality. Secondary outcome measures were 30-day and 90-day postoperative mortality, length of stay (LOS), complications, readmissions, and causes of death. The benefit of the extra medical visits was assessed based on the findings and interventions noted at each visit. Descriptive statistics and simple tests (Fisher, chi-squared, and Student’s *t*) were used.

## Results

### Patients, screening, and inclusion

From 4 March, 2014, to 7 October, 2016, 673 patients were screened and 326 (48%) fulfilled one or more of the criteria in Table 1. Of these, 179 (55%) had a cardiovascular disorder, 225 a lung condition (69%), and 79 (24%) had both. One screening form was lost and could not be retrieved.

Of the 326 patients, 16 were not included due to the inability to comprehend the information (8) or disseminated and/or concurrent malignant disease with limited life expectancy (8). A total of 108 patients declined participation, mainly because of the inconvenience of the extra visits.

Four of 202 included patients were excluded before randomisation due to cancelled operation (2), withdrawal of consent (1), or disseminated disease (1). Thus, 198 patients were randomised, 99 to the IG, and 99 to the SG. Two patients in the IG withdrew their consent after randomisation. A further 13 patients later declined the extra visits in the IG but agreed to remain in the study for an intention-to-treat analysis. Two patients were unexpectedly found to be incurable at the operation, but remained in the study.

The 347 patients screened negative constitute our background population. One patient, who had two operations within three months, was screened twice and therefore excluded, leaving 346 patients for analysis.

Baseline and screening data are shown in Tables 1 and 2.

**Table 2:** Baseline characteristics of 672 patients screened for cardiopulmonary comorbidity before planned colorectal cancer surgery

	Screened negative (*n* = 346)	Screened positive (*n* = 326)				
		All 326 patients	Cardiovascular criteria (*n* = 179)	Pulmonary criteria (*n* = 225)	Included and evaluable (*n* = 196)	
					Standard group (*n* = 99)	Intervention group (*n* = 97)
Age (years) (median, range)	69 18–91	71.5 44–92	74 44–92	71 47–91	70 47–92	70 52–86
Male, *n* (%)	181 (52%)	192 (59%)	112 (63%)	137 (61 %)	64 (65 %)	61 (63%)
BMI (kg/m^2^) (median, range) (non-missing)	Not available	26.3 (15.9–45) (323)	26.7 (17.9–42.6) (177)	25.9 (15.9–45) (224)	27.1 (18.5–45) (99)	26.8 (17.9–39.4) (97)
Tumour site, *n* (%)						
Colon	223 (64%)	234 (72%)	126 (70%)	164 (73 %)	68 (69 %)	72 (74%)
Rectum	123 (36%)	92 (28%)	53 (30%)	61 (27 %)	31 (31 %)	25 (26%)
Pathoanatomy, *n* (%)						
Colorectal cancer						
-Stage I	65 (19%)	81 (25%)	42 (23%)	59 (26%)	19 (19%)	28 (29%)
-Stage II	96 (28%)	96 (29%)	57 (32%)	73 (32%)	37 (37%)	29 (30%)
-Stage III	112 (32%)	87 (27%)	48 (27%)	54 (24%)	27 (27%)	22 (23%)
-Stage IV	29 (8%)	28 (9%)	16 (9%)	16 (7%)	7 (7%)	9 (9%)
-CPR^1^	1 (0%)	0	0	0	0	0
Benign	39 (11%)	30 (9%)	12 (7%)	23 (10%)	9 (9%)	8 (8%)
Other malignancy	1 (0%)	2 (1%)	2 (1%)	0	0	1 (1%)
Unknown	3 (1%)	2 (1%)	2 (1%)	0	0	0
Surgical access						
Laparoscopic/robot					66 (67%)	66 (68%)
Open/converted					33 (33%)	31 (32%)
ASA score, *n* (%)						
1		25 (8%)			6 (6%)	7 (7%)
2		155 (48%)			54 (55%)	47 (48%)
3		137 (42%)			37 (37%)	43 (44%)
4		6 (2%)			2 (2%)	0
Missing		3 (1%)			0	0
Data from NBCD^2^	(*n* = 293, 85%)	(*n* = 275, 84%)			(*n* = 84, 85%)	(*n* = 86, 89%)
CCI by NBCD						
CCI 0	216 (74%)	114 (41%)			38 (45%)	31 (36%)
CCI 1	31 (11%)	82 (30%)			27 (32%)	29 (34%)
CCI 2	23 (8%)	37 (13%)			7 (8%)	14 (16%)
CCI 3+	23 (8%)	42 (15%)			12 (14%)	12 (14%)
WHO PS by NBCD						
PS 0	243 (83%)	177 (64%)			61 (73%)	55 (64%)
PS 1	38 (13%)	58 (21%)			15 (18%)	22 (26%)
PS 2	7 (2%)	30 (11%)			7 (8%)	7 (8%)
PS 3	1 (0%)	6 (2%)			1 (1%)	1 (1%)
PS 4	1 (0%)	1 (0%)			0	0
Unknown	3 (1%)	3 (1%)			0	1 (1%)

^1^Complete pathological response

^2^Data on Charlson Comorbidity Index (CCI) and WHO Performance Status (PS) retrospectively available from the National Bowel Cancer Database (NBCD)

### Preoperative visits

Of the 108 evaluable patients screened positive for cardiovascular conditions 107 (99%) had a preoperative visit at the CC whereas one had been seen by a cardiologist five months earlier and was not referred again. Ninety percent of the visits took place within 10 days before surgery. In 19 of these 97 cases (20%), a previously undiagnosed cardiovascular disorder was found (mostly valve disorders and hypertensive cardiac disease). In 28 cases (29%), the cardiology visit led to change of medication (26) or other intervention (2). This applies to nine of the 19 patients with newly diagnosed disease. No operations were postponed or cancelled.

The preoperative visit at the RMC was completed by 137 (99%) of the 138 evaluable patients screened positive for pulmonary disorders; one had missing information. Ninety percent of the visits took place within 10 days before the operation. In 32 of these 124 cases (26%), a previously undiagnosed pulmonary disorder was found (obstructive disease in all cases), and in 33 patients (27%), the visit led to a change in medication. This applies to 21 (66%) of the patients with newly diagnosed disease. No operations were postponed or cancelled.

### Planned postoperative medical visits (Table 3)

Of the 97 patients randomised to the IG, 93 (96%) were seen as planned by a physician before discharge from the hospital. In four patients, the visit was missed due to clerical or communication error.

**Table 3:** In-hospital events after colorectal cancer surgery in the 196 randomised and evaluable patients

	Standard group (*n* = 99)	Intervention group (*n* = 97)
Findings at planned postoperative visit		
Heart failure		3
Arrhythmia		2
Pneumonia		4
Worsening of COLD		6
Other medical event		7
Severity		
MESS^1^ 0		7
MESS 1		11
MESS 2		4
Medical complications		
Stroke	2	–
Aspiration	1	–
Pneumonia	9	8
Heart failure	2	4
Arrhythmia	2	5
Pulmonary embolism	1	–
Lung failure	4	5
Sepsis	8	5
Other	2	5
MESS 2+, all causes	10	11
Clavien 3+, all causes	7	6
Surgical complications		
Bleeding	9	4
Burst abdomen	1	3
Obstruction, ileus	4	2
Wound abscess	2	–
Intraabd. abscess	3	1
Stoma complication	–	2
Anastomotic leakage	4	9
Other	2	2
Clavien 3+, all causes	13	18
Unplanned reoperation	10	13
Unplanned return to ICU	7	8
Unplanned stay in other department	6	2

^1^Medical Event Severity Score, see text for the explanation

A reintervention under general anaesthesia before the planned postoperative visit was performed in five of the 93 patients (four patients once and one patient twice). Their postoperative visits were postponed to at least 4 days after the last reoperation, which was four, six, and 16 days after the primary operation in one, three, and one patient, respectively. Of the 88 patients without reinterventions, one was seen on POD3, 75 on POD4, 10 on POD5, and two on POD7.

Significant cardiac and pulmonary events were seen in four (4%) and 10 (11%) patients, respectively. Other significant medical problems were found in seven patients (8%). Details are given in Table 3. In all, 20 patients (22%) were found to have a new or worsened medical problem. One patient had three problems, and 19 had one problem. Of these 22 problems, nine had been identified before the visit but not treated adequately, and eight were discovered at the visit. In 11 cases, only a change in medication was needed, and in four cases another specialist was summoned.

### In-hospital complications

In-hospital medical complications are shown in Table 3. One patient who aspirated and sustained a cardiac arrest during the first postoperative night was classified as aspiration, not arrhythmia (Clavien 4b). One patient developed asystole during induction of anaesthesia but was immediately resuscitated. The operation was successfully completed 4 days later after implantation of a pacemaker, and the complication was classified as arrhythmia (Clavien 4a) possibly elicited by an allergic reaction. Overall, 22 patients (22%) in the SG had a medical complication vs. 19 (20%) in the IG. Surgical complications occurred in 22 (22%) and 19 (20%) patients in the SG and IG, respectively (Table 3).

### Length of stay and short-term mortality (Table 4)

LOS is presented separately for colon and rectum cancer patients with no difference between the randomisation groups. There were no in-hospital or short-term deaths in either of the randomisation groups. Data on non-eligible patients and those who declined participation are shown for comparison.

**Table 4:** Length of stay and mortality after colorectal cancer surgery

	Standard group (*n* = 99)	Intervention group (*n* = 97)	Eligible declined (*n* = 104)^1^	Non-eligible (*n* = 332)^2^
Length of stay, colon				
Mean	7.41	7.94	6.48	5.73
95% CI	6.19–8.63	6.56–9.33	5.35–7.61	5.13–6.34
Median	6	6	5	4
Range	2–31	3–43	2–22	1–34
Length of stay, rectum				
Mean	13.52	12.48	10.59	10.38
95% CI	9.34–17.70	9.13–15.83	7.84–13.33	8.81–11.95
Median	9	10	7	7
Range	2–41	3–37	2–30	3–44
30-day mortality	0	0	4 (4%)	3 (1%)
90-day mortality	0	0	5 (5%)	3 (1%)
1-year mortality	2 (2%)	3 (3%)	7 (7%)	11 (3%)

^1^Four not resected at Vejle Hospital

^2^14 not resected at Vejle Hospital

### Follow-up visits

As stated above, 13 patients declined at an early stage to have extra follow-up visits. In addition, one patient missed the follow-up visit after 1 month and two patients their 3-month follow-up in the RMC. Thus, 1 month postoperatively, 83 of the 97 patients (86%) in the IG were seen in the RMC and 84 (87%) in the CC. Three months after the operation 82 of 97 patients (85%) were seen in the RMC.

The first RMC follow-up visit took place a median of 33 days after the operation (90% within 25–48 days). In five patients, a worsening of pre-existing lung disease was found, and in another five, a new pulmonary problem had arisen since the operation or was disclosed at the visit. In four patients, the new problem led to a change in medication or other intervention. Overall, 13 of the 83 patients (16%) had a change in medication or other intervention.

The CC follow-up visit took place a median of 33 days after the operation (90% within 26–50 days). In one case, a worsening of pre-existing heart disease led to further outpatient visits. In four patients, a new cardiac disorder had supervened since the operation or was disclosed at the visit. Overall, 13 of the 84 patients (15%) had a change in medication or other intervention.

The second RMC visit took place a median of 93 days after the operation (90% within 84–110 days). In eight patients, a worsening of pre-existing lung disease was noted, and in 13 cases, a new pulmonary problem had occurred since the operation. Overall, 19 patients (23%) had a change in medication or other intervention at the visit.

### Long-term outcomes (Tables 4 and 5 )

Significantly less IG than SG patients had one or more unplanned readmissions within 90 days after surgery (16 and 30, respectively, *p* = 0.028, Fisher). Readmissions were significantly related to in-hospital complications (*p* = 0.007, Fisher). Readmissions with medical complications were more common in patients discharged with an ileostomy (*p* = 0.029, Fisher). The distribution of stomas, however, did not differ between randomisation groups. One year after the operation, two patients in the SG and three in the IG had died; one died of unknown causes and four had developed terminal cancer and died in hospital (1), hospice (1), or their home (2).

**Table 5:** Unplanned readmissions with complications after colorectal surgery

	Standard group(*n* = 99)	Intervention group (*n* = 97)
Readmissions with medical complications		
Patients, *n* (%)	16 (16%)	10 (10%)
No. of readmissions		
1	14	7
2	1	3
3	1	–
Complications^1^		
Stroke	1	–
ACS	–	1
Pneumonia	2	1
New arrhythmia	2	1
Pulmonary embolism	–	1
Kidney failure	–	2
Other		
-Angioedema	1	–
-Dehydration/electrolyte	5	3
-Nausea	2	–
-Syncope/fall	1	1
-Diarrhoea	1	1
-Ataxia	1	–
-Chest pain	2	1
-Fever, no focus	–	1
Clavien 3+, all causes	1	1
Readmissions with surgical complications		
Patients, *n* (%)	17 (17%)	8 (8%)
No. of readmissions		
1	13	6
2	3	1
3	–	1
5	1	–
Complications^1^		
Bleeding	1	1
Intraabdominal abscess	1	2
Anastomotic leakage	3	1
Other		
-High output/diarrhoea	1	1
-Urinary tract	4	–
-Stoma blockage/constipation	5	2
-Flap dehiscence	1	–
-Abdominal/perineal pain	3	–
-Late bowel perforation	–	1
Clavien 3+, all causes	5	4

^1^Main reasons for readmission, may not sum up to total number of readmissions

### Assessment of bias (Table 2)

To test for inadvertent selection of the most healthy and self-sufficient patients for inclusion, we compared the WHO performance status (PS) and the Charlson Comorbidity Index (CCI) automatically calculated in the NBCD between the eligible patients declining participation and the included patients and found no significant differences. Data were available in 568 of 672 patients (85 %), the proportion of which did not differ between the groups (Table 2).

Baseline characteristics were evenly distributed between the randomisation groups, except for the proportion of patients fulfilling cardiovascular eligibility criteria, which was higher in the IG than in the SG (62 (64%) vs. 46 (46%), *p* = 0.015, Fisher) whereas the presence of pulmonary criteria was not significantly different (65 (67%) vs.73 (74%)). There was no significant relationship between these criteria and readmissions.

We also compared the two randomisation groups with respect to pre- and postoperative oncological treatment and liver or lung surgery, from 6 months before to 6 months after the index operation. The groups did not differ regarding preoperative chemotherapy (SG, 19%, IG 10%), postoperative chemotherapy (SG, 33%; IG, 28%), postoperative radiotherapy (SG, 2; IG, 0), lung surgery (SG, 2; IG, 2), preoperative liver surgery (SG, 0; IG, 1), or postoperative liver surgery (SG, 4; IG, 5). A larger proportion of patients in the SG than in the IG received preoperative radiotherapy (pRad) (14% vs. 5%, *p* = 0.051, Fisher), and pRad was significantly associated with readmission. Two of these patients, however (one in each group), had pRad for other diseases (breast and pharynx cancer). After excluding all patients with pRad, the readmission rate was still higher in the SG than in the IG (27% vs. 15%), but the difference did not reach statistical significance (*p* = 0.065, Fisher). The other treatment modalities were not associated with readmission.

## Discussion

This study aimed to test a trial setup for assessing the effect of extra postoperative medical visits on the outcome of colorectal cancer surgery and to obtain reliable estimates of the outcome measures in the current setting.

We succeeded in screening all relevant patients and treating nearly all included patients as planned during the preoperative and in-hospital phases, but 15% missed one or both planned postoperative outpatient visits. Also, a third of the eligible patients declined study participation.

The extra in-hospital visits disclosed new or worsened medical problems in 22% of the patients often leading to a change in medication or involvement of other specialists. At the extra outpatient visits, changes in medication or other actions were taken in 15–23% of the patients, but these extra efforts in the IG did not translate into improved survival.

The 1-year mortality was only 2.6% among the included patients, with no difference between the two randomisation groups, and there were no deaths within the first 90 days after the operation. Moreover, nearly all deaths within the first year after the operation could be ascribed to malignancy rather than comorbidity.

Previous population-based studies have reported substantially higher 30-day and 1-year mortality rates after elective colorectal surgery [2, 5], but postoperative mortality has decreased markedly during the last decade [30, 31]. Our findings compare well to recent Danish national data [22].

A few previous studies have tried to elucidate causes of death within the first year after colorectal cancer surgery and found that most patients died directly or indirectly of colorectal cancer, except the elderly ≥ 75 years of age [32–35].

The secondary outcomes of LOS and rate of in-hospital complications did not differ significantly between the randomisation groups, but we found significantly fewer readmissions in the IG. In previous studies, LOS and readmissions were associated with comorbidity [36–38] but also with postoperative complications [39, 40]. LOS in our patients differed between rectal and colonic cases, but this was also found in previous reports, particularly in rectal cancer patients with a stoma [36–38]. Our 90-day readmission rate of 23% compares well to previous series with an LOS comparable to ours [41].

Particular strengths of this study are its prospective, randomised design and its real-life setting in an average-sized hospital with patients recruited based on simple criteria from an otherwise unselected, consecutive series of elective cases. Also, thanks to common EMRs and administrative databases we could track 90-day readmissions and complications effectively and account for pre- and postoperative adjuvant treatment as well as causes of death. The main limitation of the study is its small size, which was, however, a deliberate choice. As it turned out, mortality in the study group was much lower than expected, making it impossible to show any difference between the groups regarding the primary outcome measure. Two potential confounders were unevenly distributed between the randomisation groups, probably owing to the small number of patients. There were relatively more patients with cardiovascular disease in the IG but at the same time fewer readmissions and this would speak in favour of the intervention as a means to reduce readmissions. On the other hand, relatively more patients in the SG had preoperative radiotherapy, and this was associated with readmissions. Excluding irradiated patients from the analysis still resulted in a 44% lower readmission rate in the IG than in the SG. Furthermore, any effect of the postoperative interventions may potentially have been attenuated by the planned preoperative visits, but for obvious ethical reasons, these could not be withheld from the patients. The fact that 35% of the eligible patients did not wish to participate and 15% in the intervention group declined some of the planned follow-up is also a limitation but it reflects the real-life setting of the study and prevents overrating the benefit of the intervention.

We suggest that future studies should focus on interventions directed against postoperative complications and readmissions, which are still common.

## Conclusion

We found a low rate of postoperative mortality after elective surgery for colorectal cancer even in patients with cardiopulmonary risk factors. There was no evidence of reduced mortality with additional medical follow-up in these patients.

## Data Availability

The datasets generated and analysed during the current study are not publicly available in accordance with Danish data protection legislation. The corresponding author will try to meet any reasonable request, provided that prior permission can be obtained from Danish Data Protection Agency.

## Abbreviations

ACS: Acute coronary syndrome
ASA: American Society of Anesthesiologists
CC: Cardiology Clinic
CCI: Charlson Comorbidity Index
CPR: Central Office of Civil Registration
CRC: Colorectal cancer
DCCG: Danish Colorectal Cancer Group
DVT: Deep vein thrombosis
EMR: Electronic medical record
ERAS: Enhanced Recovery After Surgery
ICU: Intensive care unit
LOS: Length of stay
LPR: National Patient Registry
LRP: National Pathology Registry
MESS: Medical Event Severity Score
NBCD: National Bowel Cancer Database
POD: Postoperative day
PS: WHO Performance Status
RMC: Respiratory Medicine Clinic

## References

[1] Boakye D, Rillmann B, Walter V, Jansen L, Hoffmeister M, Brenner H (2018). Impact of comorbidity and frailty on prognosis in colorectal cancer patients: a systematic review and meta-analysis. Cancer Treatment Rev..

[2] Iversen LH (2012). Aspects of survival from colorectal cancer in Denmark. Dan Med J..

[3] Ostenfeld EB, Norgaard M, Thomsen RW, Iversen LH, Jacobsen JB, Sogaard M (2013). Comorbidity and survival of Danish patients with colon and rectal cancer from 2000-2011: a population-based cohort study. Clin Epidemiol..

[4] Gooiker GA, Dekker JW, Bastiaannet E, van der Geest LG, Merkus JW, van de Velde CJ (2012). Risk factors for excess mortality in the first year after curative surgery for colorectal cancer. Ann Surg Oncol..

[5] Wu CC, Hsu TW, Chang CM, Yu CH, Lee CC (2015). Age-adjusted Charlson comorbidity index scores as predictor of survival in colorectal cancer patients who underwent surgical resection and chemoradiation. Medicine..

[6] Degett TH, Roikjaer O, Iversen LH, Gogenur I (2018). A model predicting operative mortality in the UK has only limited value in Denmark. Int J Colorectal Dis..

[7] Hu WH, Chen HH, Lee KC, Liu L, Eisenstein S, Parry L (2016). Assessment of the addition of hypoalbuminemia to ACS-NSQIP surgical risk calculator in colorectal cancer. Medicine..

[8] Kong CH, Guest GD, Stupart DA, Faragher IG, Chan ST, Watters DA (2015). Colorectal preOperative Surgical Score (CrOSS) for mortality in major colorectal surgery. ANZ J Surg..

[9] Bowles TA, Sanders KM, Colson M, Watters DA (2008). Simplified risk stratification in elective colorectal surgery. ANZ J Surg..

[10] Haga Y, Wada Y, Ikenaga M, Takeuchi H, Ikejiri K (2011). Evaluation of modified estimation of physiologic ability and surgical stress in colorectal carcinoma surgery. Dis Colon Rectum..

[11] Moran J, Wilson F, Guinan E, McCormick P, Hussey J, Moriarty J (2016). Role of cardiopulmonary exercise testing as a risk-assessment method in patients undergoing intra-abdominal surgery: a systematic review. Br J Anaesth..

[12] Fagard K, Leonard S, Deschodt M, Devriendt E, Wolthuis A, Prenen H (2016). The impact of frailty on postoperative outcomes in individuals aged 65 and over undergoing elective surgery for colorectal cancer: a systematic review. J Geriat Oncol..

[13] West MA, Parry MG, Lythgoe D, Barben CP, Kemp GJ, Grocott MP (2014). Cardiopulmonary exercise testing for the prediction of morbidity risk after rectal cancer surgery. Br J Surgery..

[14] McIsaac DI, Huang A, Wong CA, Wijeysundera DN, Bryson GL, van Walraven C (2017). Effect of preoperative geriatric evaluation on outcomes after elective surgery: a population-based study. J Am Geriatr Soc..

[15] Partridge JS, Harari D, Martin FC, Peacock JL, Bell R, Mohammed A (2017). Randomized clinical trial of comprehensive geriatric assessment and optimization in vascular surgery. Br J Surg..

[16] Rivera RA, Nguyen MT, Martinez-Osorio JI, McNeill MF, Ali SK, Mansi IA (2012). Preoperative medical consultation: maximizing its benefits. Am J Surg..

[17] Fleisher LA, Fleischmann KE, Auerbach AD, Barnason SA, Beckman JA, Bozkurt B (2014). 2014 ACC/AHA guideline on perioperative cardiovascular evaluation and management of patients undergoing noncardiac surgery: a report of the American College of Cardiology/American Heart Association Task Force on practice guidelines. J Am Coll Cardiol..

[18] Riggs KR, Segal JB (2016). What is the rationale for preoperative medical evaluations? A closer look at surgical risk and common terminology. Br J Anaesth..

[19] Groot MW, Spronk A, Hoeks SE, Stolker RJ, van Lier F (2017). The preoperative cardiology consultation: indications and risk modification. Neth Heart J..

[20] Leeds IL, Canner JK, Gani F, Meyers PM, Haut ER, Efron JE, et al. Increased healthcare utilization for medical comorbidities prior to surgery improves postoperative outcomes. Ann Surg. 2018. 10.1097/SLA.0000000000002851. 10.1097/SLA.0000000000002851 PMC8559326 29864092

[21] Gillis C, Buhler K, Bresee L, Carli F, Gramlich L, Culos-Reed N (2018). Effects of nutritional prehabilitation, with and without exercise, on outcomes of patients who undergo colorectal surgery: a systematic review and meta-analysis. Gastroenterology..

[22] Ingeholm P, Eriksen MR, Cueto HTØ, Njor SH (2016). Danish Colorectal Cancer Group: Annual Report 2016.

[23] Platon AM, Erichsen R, Christiansen CF, Andersen LK, Svaerke C, Montomoli J (2014). The impact of chronic obstructive pulmonary disease on intensive care unit admission and 30-day mortality in patients undergoing colorectal cancer surgery: a Danish population-based cohort study. BMJ Open Resp Res..

[24] Larsen SHV, L.D.; Krarup, N.H.V. [Cardiac risk assessment before non-cardiac surgery. National guideline (in Danish).]. Danish Society on Cardiology (DCS). 2013.

[25] Fletcher CM (1952). The clinical diagnosis of pulmonary emphysema; an experimental study. Proc Royal Soc Med..

[26] Clavien PA, Barkun J, de Oliveira ML, Vauthey JN, Dindo D, Schulick RD (2009). The Clavien-Dindo classification of surgical complications: five-year experience. Annals of surgery..

[27] Dindo D, Demartines N, Clavien PA (2004). Classification of surgical complications: a new proposal with evaluation in a cohort of 6336 patients and results of a survey. Ann Surg..

[28] Ingeholm P, Gogenur I, Iversen LH (2016). Danish Colorectal Cancer Group Database. Clin Epidemiol..

[29] Moher D, Hopewell S, Schulz KF, Montori V, Gotzsche PC, Devereaux PJ (2010). CONSORT 2010 explanation and elaboration: updated guidelines for reporting parallel group randomised trials. BMJ..

[30] Iversen LH, Green A, Ingeholm P, Osterlind K, Gogenur I (2016). Improved survival of colorectal cancer in Denmark during 2001-2012 - the efforts of several national initiatives. Acta Oncol..

[31] Rutegard M, Haapamaki M, Matthiessen P, Rutegard J (2014). Early postoperative mortality after surgery for rectal cancer in Sweden, 2000-2011. Colorectal Dis..

[32] Dekker JW, Gooiker GA, Bastiaannet E, van den Broek CB, van der Geest LG, van de Velde CJ (2014). Cause of death the first year after curative colorectal cancer surgery; a prolonged impact of the surgery in elderly colorectal cancer patients. Eur J Surg Oncol..

[33] Kornmann VNN, van Vugt JLA, Smits AB, van Ramshorst B, Boerma D (2017). The first year after colorectal surgery in the elderly. Ann Coloproctol..

[34] Makela JT, Klintrup KH, Rautio TT (2017). Mortality and survival after surgical treatment of colorectal cancer in patients aged over 80 years. Gastrointestinal Tumors..

[35] Souwer ETD, Bastiaannet E, de Bruijn S, Breugom AJ, van den Bos F, Portielje JE (2018). Comprehensive multidisciplinary care program for elderly colorectal cancer patients: “from Prehabilitation to Independence”. Eur J Surg Oncol..

[36] Faiz O, Haji A, Burns E, Bottle A, Kennedy R, Aylin P (2011). Hospital stay amongst patients undergoing major elective colorectal surgery: predicting prolonged stay and readmissions in NHS hospitals. Colorectal Dis..

[37] Kelly M, Sharp L, Dwane F, Kelleher T, Comber H (2012). Factors predicting hospital length-of-stay and readmission after colorectal resection: a population-based study of elective and emergency admissions. BMC Health Services Res..

[38] Pucciarelli S, Zorzi M, Gennaro N, Gagliardi G, Restivo A, Saugo M (2017). In-hospital mortality, 30-day readmission, and length of hospital stay after surgery for primary colorectal cancer: a national population-based study. Eur J Surg Oncol..

[39] Keller DS, Bankwitz B, Woconish D, Champagne BJ, Reynolds HL, Stein SL (2014). Predicting who will fail early discharge after laparoscopic colorectal surgery with an established enhanced recovery pathway. Surg Endosc..

[40] Krarup PM, Nordholm-Carstensen A, Jorgensen LN, Harling H (2015). Association of comorbidity with anastomotic leak, 30-day mortality, and length of stay in elective surgery for colonic cancer: a nationwide cohort study. Dis Colon Rectum..

[41] Wick EC, Shore AD, Hirose K, Ibrahim AM, Gearhart SL, Efron J (2011). Readmission rates and cost following colorectal surgery. Dis Colon Rectum..

